# Integration of new alternative reference strain genome sequences into the *Saccharomyces* genome database

**DOI:** 10.1093/database/baw074

**Published:** 2016-05-31

**Authors:** Giltae Song, Rama Balakrishnan, Gail Binkley, Maria C. Costanzo, Kyla Dalusag, Janos Demeter, Stacia Engel, Sage T. Hellerstedt, Kalpana Karra, Benjamin C. Hitz, Robert S. Nash, Kelley Paskov, Travis Sheppard, Marek Skrzypek, Shuai Weng, Edith Wong, J. Michael Cherry

**Affiliations:** Department of Genetics, Stanford University, Stanford, CA, USA

## Abstract

The *Saccharomyces* Genome Database (SGD; http://www.yeastgenome.org/) is the authoritative community resource for the *Saccharomyces cerevisiae* reference genome sequence and its annotation. To provide a wider scope of genetic and phenotypic variation in yeast, the genome sequences and their corresponding annotations from 11 alternative *S. cerevisiae* reference strains have been integrated into SGD. Genomic and protein sequence information for genes from these strains are now available on the Sequence and Protein tab of the corresponding Locus Summary pages. We illustrate how these genome sequences can be utilized to aid our understanding of strain-specific functional and phenotypic differences.

**Database URL:** www.yeastgenome.org

## Introduction

The genome of the budding yeast *Saccharomyces cerevisiae* was the first available complete eukaryotic genome sequence. The reference genome for *S. cerevisiae* was determined from the strain S288C. This sequence has been fully annotated and maintained at the *Saccharomyces* Genome Database (SGD) for close to two decades. SGD has served as the repository of *Saccharomyces* *cerevisiae* genomic and biological data since that time (1, 2). Although dozens of *S. cerevisiae* strain sequences have been made accessible through SGD for the utility of sequence analysis tools such as the BLAST search (3), these sequence data were not available from SGD Sequence pages and not integrated into the database due to the uncertainty associated with the data (e.g. poor sequence coverage). With the advent of high-throughput Next Generation Sequencing technology, the genomic sequence of 337 *S. cerevisiae* strains at deep sequence fold coverage has been released by several laboratories (see references 4–15). In addition, one group will soon release over a 1000 additional yeast genome sequences (e.g. The 1002 Yeast Genome Project, http://1002genomes.u-strasbg.fr/). To move forward in the era of big genomic data, SGD has integrated genome sequences and annotations from 11 strains of *S. cerevisiae* with a substantial history of use over past decades and large body of published experimental data. Users can easily access these genomes via Sequence and Strain pages, as well as through the sequence analysis tools provided by SGD.

## Integration of Alternative Reference Genome Sequences

**Figure 1.**
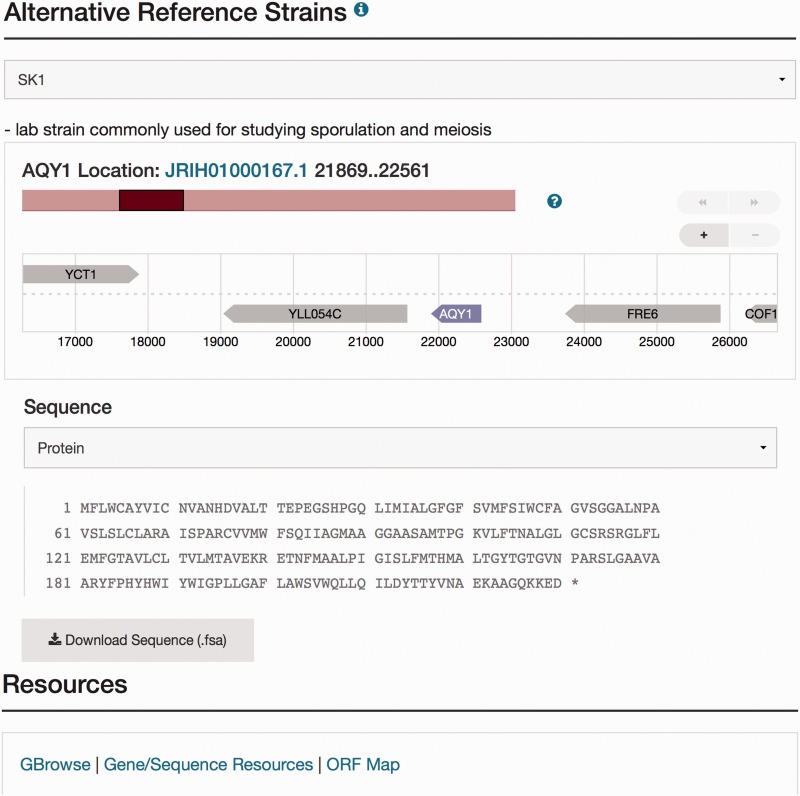
Visualization of the new sequence data for *AQY1* in the database. The Sequence page can be accessed by selecting the tab at the top of the *AQY1* LSP, labeled ‘Sequence.’ There are six sections within the Sequence page including the ‘Sequence Overview’ for descriptive information, ‘Alternative Reference Strains’ for viewing the DNA or protein sequence in a selected alternative reference strain, and ‘Resources’ for access to processed results using sequence analysis tools (e.g. a new tool called SGD Variant Viewer (28), updated BLAST search, and ClustalW multiple sequence alignments). Selection of SK1 as an alternative strain is illustrated in this figure. Neighbor genes of *AQY1* in SK1 are also shown in the visualization. When the user clicks a sequence analysis tool (URL in blue) in the resources tab, they will view the gene-specific results using that tool.

At SGD, we have incorporated these non-S288C alternative strain sequences into the database. The alternative reference strains, chosen based on the availability of substantial amounts of published experimental data, include: CEN.PK (16), D273-10B (17), FL100 (18), JK9-3d (19), RM11-1a (20), SEY6210 (21), SK1 (22), Sigma1278b (23), W303 (24), X2180-1A (25) and Y55 (26) (see Table 1). These are the genomes for which we have the most curated phenotype data, and for which we aim to curate specific functional information. For each of the 11 non-S288C sequences, there is an annotation available for each open reading frame **(**ORF) based on the annotation of the reference S288C strain. The genomic, coding and protein sequences for the ORFs in these other strains are available to view and download, on the Sequence page of the corresponding ORF in the reference strain S288C (see Figure 1). In addition to these 11 alternative strain sequences, 14 other strain genomes were processed using Automated Genome Analysis PipelinE, the automatic annotation pipeline (8), and are available for download from the Sequence page. All are accessible for sequence analysis tools provided by SGD.

**Table 1.** Summary information on the 11 alternative reference strains

Strain	Description	Source (ATCC ID)	NCBI BioSample Accession	Number of ORFs	Phenotype count per strain (%)	References
RM11-1a	A natural isolate collected from a California vineyard	UCD 2788 (UC Davis culture collection ID)	SAMN03020228	5323	2 (0.002)	(20)
Y55	Laboratory strain originally isolated from wine grapes	ATCC: 52530	SAMN03020218	5359	18 (0.015)	(26)
FL100	Laboratory strain	ATCC: 28383	SAMN03020232	5366	57 (0.046)	(18)
JK9-3d	Laboratory strain	ATCC: MYA-555	SAMN03020238	5385	111 (0.09)	(19)
CEN.PK	Laboratory strain popular for use in systems biology studies	ATCC: MYA-1108	SAMN03020234	5379	213 (0.174)	(16)
X2180-1A	S288C derivative laboratory strain	ATCC: 204504	SAMN03020236	5387	276 (0.225)	(25)
D273-10B	Lab strain used for mitochondrial studies	ATCC: 24657	SAMN03020237	5383	278 (0.227)	(17)
SEY6210	Lab strain used in studies of autophagy and protein sorting	ATCC: 96099	SAMN03020235	5400	414 (0.337)	(21)
SK1	Lab strain used for studying sporulation and meiosis	ATCC: 204720	SAMN03020220	5350	859 (0.7)	(22)
Sigma1278b	Used in pseudohyphal growth studies	ATCC: 42800	SAMN03020229	5358	2170 (1.768)	(23)
W303	Laboratory strain used for research into aging	ATCC: 20060	SAMN03020233	5397	3158 (2.573)	(24)

We chose 11 non-S288C strains as alternative reference strains based on the number of phenotype annotations curated in SGD. Phenotype studies are most frequently reported using the S288C reference strain, i.e. 84.78% of phenotypic counts in SGD are based on work in S288C. Other than S288C, the 11 alternative reference strains have been most frequently used for yeast phenotype studies. The alternative strains are used for specific areas of biology (e.g. CEN.PK for systems biology, D273-10B for mitochondrial studies, SEY6210 for autophagy and protein sorting, SK1 for sporulation and meiosis, Sigma1278b for pseudohyphal growth, and W303 for aging). The source of the sequenced strain genome is summarized with ATCC ID. The assembly and raw sequence data of each strain has been deposited in NCBI and can be found with NCBI BioSample accession numbers.

## Utility of the New Sequence Data Demonstrated by a Use-Case Example

**Figure 2.**
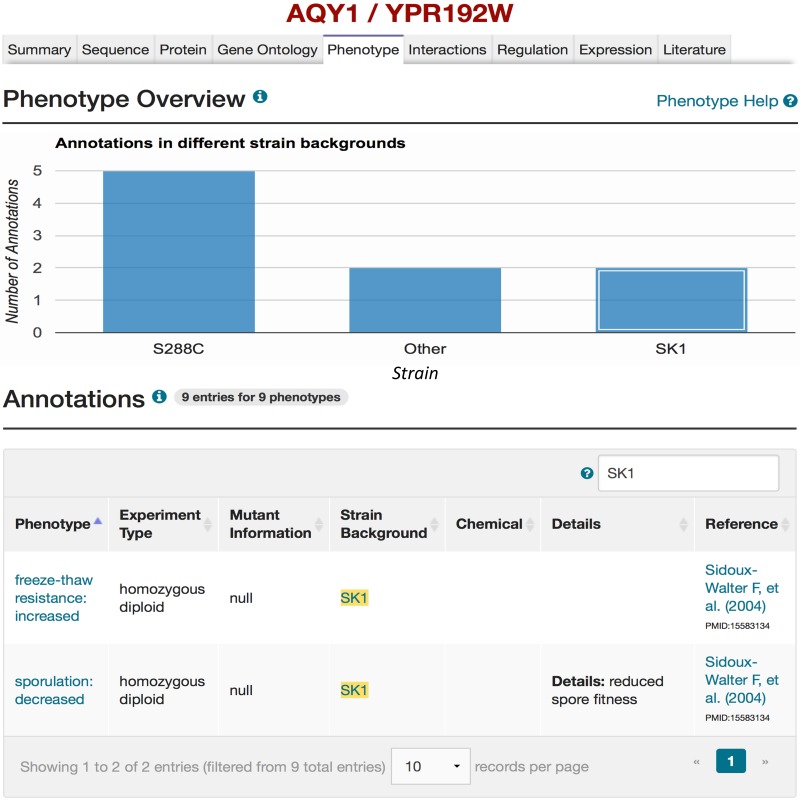
Mutant phenotypes for *AQY1* gene in SGD. By querying for ‘*AQY1’* using the SGD search box, and selecting the Phenotype tab on the *AQY1* LSP, phenotype information for *AQY1* can be viewed. A bar chart summarizes how many phenotypic annotations have been curated in different strain backgrounds (e.g. two mutant phenotypes in the SK1 strain background). If the box for SK1 in the bar chart is selected, the details of the two mutant phenotypes for *AQY1* in the SK1 strain background will be listed in the Annotations section. Users also can refer to the relevant literature (27) that describes studies on the mutant phenotypes resulted from polymorphisms in the *AQY1* gene in strain SK1 as shown in the “Reference” column of the table. Users can also access more information of the alternative strain (SK1) by selecting the strain name (highlighted in yellow) in the Annotations table (see Figure 3).

**Figure 3.**
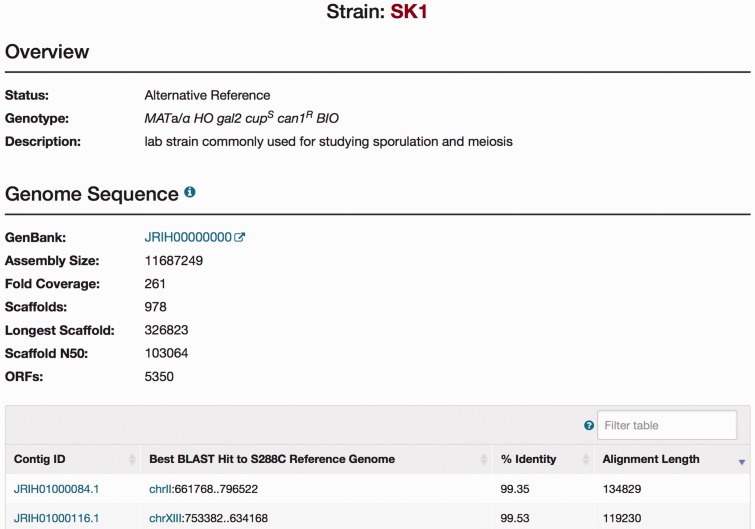
The SGD SK1 Strain page. Strain pages are available for each of the alternative strains. The Strain page includes an Overview section for descriptive information of each strain, and a Genome Sequence section for brief statistics of the new sequence integrated in the database (NCBI accession number, the number of scaffolds, and the number of ORFs) and a table for information on each contig.

In the following section, we illustrate how a researcher can use these new genome sequences in conjunction with other features in SGD. For example, if a user wishes to investigate the contribution of genetic variation in the aquaporin encoding gene *AQY1* (YPR192W) and its relevance to alterations in sporulation efficiency, represented by phenotype changes, they can start by visiting the corresponding Locus Summary Page (LSP) for *AQY1*. This page can be reached by searching for ‘AQY1’ via the quick search located at the upper right side on most pages of the SGD website (http://www.yeastgenome.org). A page with all phenotype data available for *AQY1*, accessible from the *AQY1* LSP, lists the phenotypes displayed by various mutants of *AQY1* along with relevant details such as the strain background, the type of mutant, and references (Figure 2). The bar charts available at the top of the Phenotype page summarize the breakdown of phenotypes by type of mutation and strain background. Clicking on the SK1 bar from this chart anchors to the annotation table below, providing access to the relevant annotations and associated detail (Figure 2). More information about the strain itself (i.e. genotype and assembly of the genome) can be found by selecting the strain name in the table of the Annotations section (Figure 3).

A study of sporulation-specific phenotypes within the *AQY1* gene of SK1 (27) described strain-background specific variation in two residues (V121 and P255) that contribute to the activation of *AQY1* in both SK1 and Sigma1278b. In other strain backgrounds, including S288C, *AQY1* is inactive due to mutations at these positions (M121 and T225) within the coding sequence. To explore the polymorphisms in these critical residues relevant to the phenotypic variations in all alternative strains, users can move from the Phenotype page to the Sequence page of *AQY1* by selecting the Sequence tab available at the top of the page (Figure 1). The Sequence page offers options to display the genomic and coding sequence of the gene in the reference strain S288C or in one of the alternative reference strains or other strains. If users wish to view and obtain the protein sequence of *AQY1* in SK1 strain, they can make SK1 the alternative reference strain, and simply select the protein sequence (Figure 1) from the pull-down menu. Sequences in 14 strains other than the alternative references are listed in the Other Strains section of the Sequence page where sequence is available for download. Sequence tools containing the new reference genome sequence such as BLAST and sequence alignment options with other *S. cerevisiae* and fungal sequences are accessible in the Resources section of the Sequence page. A link to Variant Viewer, a new visualization tool within SGD is also in the Resource section of the Sequence page (28).

**Figure 4.**
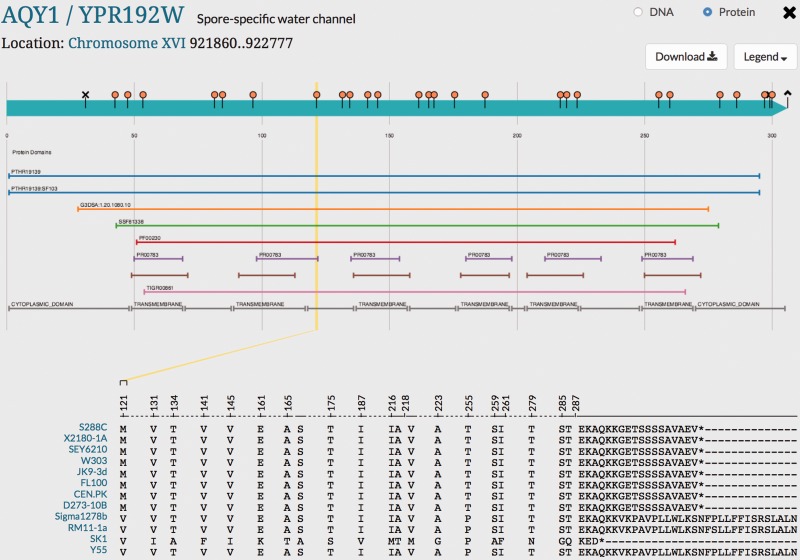
Visualization of genetic variation within *AQY1* gene across alternative reference strains in SGD. Variations within *AQY1* among 11 alternative strains and the S288C reference strain are depicted in the SGD Variant Viewer. Non-synonymous mutations and deletions/insertions with their location information in *AQY1* are shown in the viewer. We can see that *AQY1* is conserved in most laboratory strains. SK1 shows the most non-synonymous mutations relative to the S288C reference unlike the other strains. There are two common mutations that appear in four strains Sigma1278b, RM11-1a, SK1 and Y55: V121 (guided in a yellow line in the viewer) and P255. SK1 has a shorter C-terminus than other strains whereas Sigma1278b, RM11-1a and Y55 have longer C-termini than the eight laboratory strains. Other than the C-terminus and two mutations (V121 and P255), Sigma1278b, RM11-1a and Y55 show conserved amino acids with the eight laboratory strains unlike SK1.

Variant Viewer can be use to visualize variation within *AQY1* in the alternative strain genomes (Figure 4). For example, the two critical mutations located at position V121 (guided in a yellow line in Figure 4) and P255 in strains Sigma1278b, RM11-1a, SK1 and Y55 that are associated with the activation of Aqy1p can be visualized. The other eight laboratory strains show M121 and T255 in these positions, which cause the inactivation of Aqy1p in these laboratory strains (29). The Aqy1p C-terminus in eight laboratory strains is conserved while Sigma1278b, RM11-1a and Y55 show a longer C-terminus and SK1, a shorter C-terminus. The extended C-terminus of Sigma1278b is known to reduce the expression of the Aqy1p (30). We can predict that the expression of Aqy1p may also be reduced in Y55 and RM11-1a and the short C-terminus in SK1 may enhance Aqy1p expression. Other than these two residues (V121 and P255) and the extended C-terminus, Sigma1278b, RM11-1a and Y55 show strong conservation at the amino acid level with the eight laboratory strains. Unlike Sigma1278b, RM11-1a and Y55, SK1 shows more variation in *AQY1* compared with the other strains. These additional mutations in SK1 may be also relevant to *AQY1* protein function. By studying the variation within the sequence of *AQY1* in the alternative reference strains, researchers can raise several important scientific questions concerning the phenotypic variation and the relationship with sequence variation in the alternative strains. This is an example of a study on a single gene. We expect that researchers studying other yeast genes can make use of these new sequence data to study variation in a similar manner.

### Future directions

The integration of the alternative strain genomes in SGD will accelerate yeast genetics and population genomics studies by providing a user-friendly environment for the use of sequence data. To increase the accuracy of users’ studies, we plan to improve the quality of genome assemblies and annotations associated with these sequence data. As updated information becomes available and errors are corrected, they will be incorporated into future genome releases. We also anticipate expanding the reference genome panel in the future to include additional strains in order to accommodate emerging or underserved areas of study. The goal of these challenging, on-going efforts is to empower yeast research as the big genomic data era of yeasts continues to emerge.

## Funding

This work is supported by a grant from the National Human Genome Research Institute at the United States National Institutes of Health (U41 HG001315). The content is solely the responsibility of the authors and does not necessarily represent the official views of the National Human Genome Research Institute or the National Institutes of Health. The funders had no role in design, data processing, implementation, decision to publish or preparation of the article. Funding for open access fee is provided by U41 HG001315.

*Conflict of interest*. None declared.
